# Pharmacogenomics Testing in Phase I Oncology Clinical Trials: Constructive Criticism Is Warranted

**DOI:** 10.3390/cancers14051131

**Published:** 2022-02-23

**Authors:** Tristan M. Sissung, William D. Figg

**Affiliations:** Clinical Pharmacology Program, Office of the Clinical Director, National Cancer Institute, Bethesda, MD 20892, USA; sissungt@mail.nih.gov

**Keywords:** phase I clinical trial, oncology, pharmacogenomics, pharmacogenetics

## Abstract

**Simple Summary:**

Phase I clinical trials are a cornerstone of pharmaceutical development in oncology. Many studies have now attempted to incorporate pharmacogenomics into phase I studies; however, many of these studies have fundamental flaws that that preclude interpretation and application of their findings. Study populations are often small and heterogeneous with multiple disease states, multiple dose levels, and prior therapies. Genetic testing typically includes few variants in candidate genes that do no encapsulate the full range of phenotypic variability in protein function. Moreover, a plurality of these studies do not present scientifically robust clinical or preclinical justification for undertaking pharmacogenomics studies. A significant amount of progress in understanding pharmacogenomic variability has occurred since pharmacogenomics approaches first began appearing in the literature. This progress can be immediately leveraged for the vast majority of Phase I studies. The purpose of this review is to summarize the current literature pertaining to Phase I incorporation of pharmacogenomics studies, analyze potential flaws in study design, and suggest approaches that can improve design of future scientific efforts.

**Abstract:**

While over ten-thousand phase I studies are published in oncology, fewer than 1% of these studies stratify patients based on genetic variants that influence pharmacology. Pharmacogenetics-based patient stratification can improve the success of clinical trials by identifying responsive patients who have less potential to develop toxicity; however, the scientific limits imposed by phase I study designs reduce the potential for these studies to make conclusions. We compiled all phase I studies in oncology with pharmacogenetics endpoints (*n* = 84), evaluating toxicity (*n* = 42), response or PFS (*n* = 32), and pharmacokinetics (*n* = 40). Most of these studies focus on a limited number of agent classes: Topoisomerase inhibitors, antimetabolites, and anti-angiogenesis agents. Eight genotype-directed phase I studies were identified. Phase I studies consist of homogeneous populations with a variety of comorbidities, prior therapies, racial backgrounds, and other factors that confound statistical analysis of pharmacogenetics. Taken together, phase I studies analyzed herein treated small numbers of patients (median, 95% CI = 28, 24–31), evaluated few variants that are known to change phenotype, and provided little justification of pharmacogenetics hypotheses. Future studies should account for these factors during study design to optimize the success of phase I studies and to answer important scientific questions.

## 1. Introduction

For approximately 20 years, pharmacogenomics approaches have been appearing in phase I clinical trials of anticancer medications. Accounting for genetic variability in early clinical development is worthwhile for agents in which marker-based patient selection is likely to improve success by identifying responsive and lower-risk populations [1]. This is particularly true for oncology agents, which have the highest attrition rates in clinical development and are the most likely to benefit from patient stratification [2]. Yet, the scientific constraints imposed by phase I study designs also limit the usefulness of such approaches [3]. How can reproducible or generalizable results be generated in small, heterogeneous, heavily pretreated populations that are administered combinations of various medications? Can these limitations be overcome to produce robust clinical analyses accounting for genetic variation in dose optimization? Constructive criticism of published phase I trials incorporating pharmacogenomics is warranted, and many lessons can be learned by examining the performance of such studies over two decades.

## 2. Preclinical and Early Clinical Development—Opportunities to Optimize Pharmacogenomics Testing

Following drug discovery, lead optimization is conducted in a limited set of molecules that undergoes testing for efficacy, pharmacokinetics (PK), and toxicity in model systems. Lead compounds are screened based on desirable properties associated with potential clinical utilization [4]. Such studies utilize information gathered at the bench to apply a given therapeutic to an appropriate cohort of patients in the clinical setting, and they are becoming increasingly precise. For example, traditional cancer cell lines are now being scrutinized for their applicability to human cancer in situ, which has resulted in improvements in the prioritization of therapeutic targets and drug molecules based on several genomic considerations [5,6,7].

Characterization of the absorption, distribution, metabolism, elimination, and activation (ADME-A) properties of compounds is also exceedingly important in preclinical characterizations of drug candidates since both the ability of a bioactive drug to reach the intended target and its toxicity depend on pharmacokinetic properties [4]. In vitro, in vivo, and in silico ADME-A screening techniques have become increasingly sophisticated, and many of these methods provide precise information about genetic variables that are associated with drug disposition [8,9]. In many cases, reverse translation of prior clinical experience can also be included in preclinical models that clarify the mechanistic basis of clinical observations [10].

Following discovery and preclinical characterization, molecules that are still suitable for human use move to the phases of drug development, including clinical testing [4]. A typical phase I study design involves escalating a dose that was previously determined in animal testing. The decision to increase or decrease dose is based on the presence or absence of severe toxicity at each dose level. This approach does not require assumptions about the dose-toxicity curve; however, it may expose certain populations to greater risk of toxicity should prior knowledge about variants that affect drug pharmacokinetics (PK) or pharmacodynamics (PD) be available [11]. Oftentimes, such knowledge is available from preclinical models or, perhaps more often, from retranslating prospective or retrospective analysis of clinical trial data. When decision-making is focused on target variability, patient specific factors, and PK/PD modeling, significant improvements in Phase III completion are observed [1]. These strategies include patient stratification early in the drug development process and marker-based patient selection [1,12]. Thus, appropriate application of knowledge in early clinical development reduces negative impacts on patients while simultaneously improving the attrition rate of medications undergoing development.

Despite the narrow therapeutic index of anticancer agents and the frequent need to administer these medications at high dose to avoid inefficacy, pharmacogenetic approaches are rare in the early development of oncology agents. Sufficiently powered studies with adequate genetic coverage in appropriate populations are even rarer. Why do so few studies incorporate pharmacogenetics approaches in Phase I designs, and why do so many of these studies fail to detect an association? [3] The purpose of this review is to provide an overview of currently published phase I studies incorporating germline pharmacogenomics approaches and explore the potential for improving pharmacogenomics strategies in future phase I studies.

## 3. Methods

Using “Clinical Trial, Phase I” filter in https://pubmed.ncbi.nlm.nih.gov, we searched for the following terms: “pharmacogenetics cancer”, “pharmacogenomics cancer”, “polymorphism cancer”, “pharmacogenetics leukemia”, “pharmacogenomics leukemia”, “pharmacogenetics oncology”, “pharmacogenomics oncology”, and “polymorphism oncology”. The final search for these studies was conducted on 21 January 2022. Studies were included if they contained data about at least one commonly inherited germline genetic variant. Studies were excluded if they only pertained to cancer mutations (i.e., companion diagnostics) and/or gene expression. Of 11,737 phase I clinical trials published on the subject of “cancer”, and 14,247 phase I clinical trials mentioning “oncology”, we found only 84 different phase I, phase Ib, and phase I/II clinical trials that met the above criteria (0.72% and 0.59% of studies, respectively). All studies utilized the candidate gene approach, and no study included hypothesis-free methods. The present analysis includes studies regardless of prospective or retrospective design provided a gene–drug pair was tested in a cohort of patients participating in phase I clinical testing of an anticancer agent. Characteristics of the studies are presented in Table 1.

## 4. Phase I Study Endpoints Incorporating Pharmacogenomics Testing

### 4.1. Studies Incorporating Pharmacogenomics Analysis vs. Toxicity, Response, and/or Progression-Free Survival (PFS)

**Figure 1:**
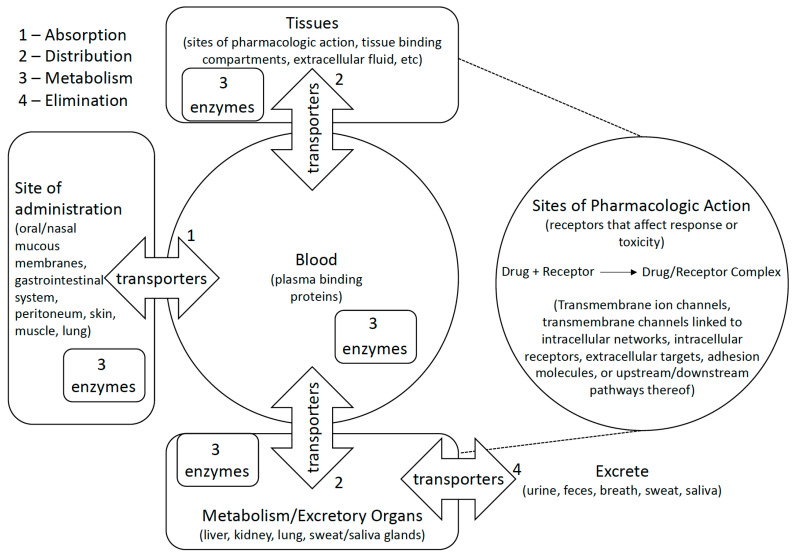
Polymorphic metabolic enzymes affect pharmacokinetics and pharmacodynamics of medications by activating/inactivating them and encouraging their elimination. Transporters similarly affect pharmacokinetics and pharmacodynamics by encouraging or preventing distribution of compounds to or from bodily compartments. Some studies examine how genetic variation affects medications at their site of pharmacologic action by studying direct or indirect effects of drug action on biological pathways.

More phase I studies we examined have compared genetic variants to drug toxicity than any other endpoint (*n* = 116 comparisons in 42 studies), and every one of these studies evaluated genes involved in the ADME-A or activity pathway of drugs under study (Figure 1). For example, the most frequent genes studied versus toxicity include UDP-glucuronosyltransferases (UGTs) that conjugate glucuronides to a variety of medications (*n* = 21 comparisons with genotype) and ATP-binding cassette transporters (ABCs) that convey several drug types across biological membranes (*n* = 15 comparisons; Table 2). As expected, fewer studies have evaluated pathways that are related to specific classes of drugs, such as the relationship between variants in *Aurora Kinase A* and *B* (*AURKA* and *AURKB*) and the AURK inhibitor, danusertib (*n* = 1 study). Studies of genetics versus response or PFS are rarer (*n* = 73 comparisons in 31 studies), but they also pertain to a mixture of genes involved in both pharmacokinetics and pharmacodynamics.

Thirteen of the 42 pharmacogenetics studies involving toxicity did not conduct a formal statistical analysis, and 11 of 32 studies related to response or PFS pharmacogenetics did not analyze data they collected (Table 2). Of the remaining 29 pharmacogenetics studies evaluating toxicity, only seven studies found an association with toxicity (18 comparisons) and 22 studies found no association (72 total comparisons). In general, low coverage was observed within each gene (median = 1; range 1–5) in few patients (median 24.5; range 10–111) at multiple dose or treatment levels (median 3 dose or treatment levels; range 1–13 levels). Of those studies analyzing response or PFS, nine of 21 studies detected an association with a genetic variant (11 comparisons) and 12 did not (47 comparisons). A median of 1 variant was detected in each gene (range 1–30) in a median of 30 patients (range 10–115) at a median of 3 dose or treatment levels (range 1–12).

### 4.2. Studies Incorporating Pharmacogenomics Analysis vs. Pharmacokinetics

Of those studies that have evaluated genetic variants in ADME-A genes or genes involved in drug action (Table 2), a median of two variants were probed per gene (range 1–61 variants). Only three studies evaluated more than 10 variants in genes involved in Phase I or II metabolism [13,14,15]. Yet, moderate to definitive evidence exists for at least 16 star alleles in *CYP2A6*, seven in *CYP2C19*, 20 in *CYP2C9*, 26 in *CYP2D6*, six in *CYP3A4*, three in *CYP3A5*, 16 in *NAT2*, and five in *UGT1A1* according to pharmgkb.org. Moreover, the genotype-predicted activity status (e.g., ultrarapid, rapid, extensive, intermediate, or poor metabolizer) of most of these genes is now available, but this information is not being used routinely in phase I studies (Table 2).

Twenty of the 40 studies that compared genotype to pharmacokinetics never conducted a formal statistical analysis (data for one study were not disclosed), instead offering an observational commentary about specific patients harboring certain genetic variants (Table 2). Of the remaining 20 studies, 13 (61.9%) found a relationship between a genetic variant and the pharmacokinetic properties of a medication (15 comparisons with genotype) and seven studies did not (43 comparisons). Of these, five studies pertained to the relationship between irinotecan (or SN38) and *UGT1A1* variants, a gene–drug interaction that is well characterized in the scientific literature with multiple iterations of retranslation [16]. A median of 28 patients were included in these studies (range 10–94) at a median of three different doses or treatments (range 1–12).

### 4.3. Critical Analysis of Phase I Study Designs Examining Toxicity, Response/PFS, and Pharmacokinetics

Studies examining the statistical relationship between pharmacokinetics and genotype demonstrate a higher ratio of statistical associations per comparison (14/59 comparisons with genotype, 23.7%) than those focused on toxicity (18/93 comparisons, 19.4%) or response/PFS (11/67 comparisons, 16.4%; Table 2), although the difference in these ratios was not statistically significant (*p* = 0.59, chi-squared test). If all endpoints are considered together, a statistically significant relationship is apparent between a higher number of patients tested and detection of an association with genotype (median = 28 patients in non-associations, median = 34 patients in associations; *p* = 0.020; Wilcoxon rank sum test). Statistical positivity in toxicity studies was also associated with the number of patients tested when these studies were considered alone (*p* = 0.014; median = 27 patients in non-associations and 34 in association; *n* = 75 and 18 studies respectively). Patient numbers were not associated with studies concerned with PK or PFS/response (*p* > 0.66). No association was detected when the number of variants tested was compared to studies that demonstrated a statistical finding (*p* = 0.61; Wilcoxon rank sum test). However, numerous genes were studied, which likely confounded the analysis. The limited number of studies per gene prevented analysis of the number of variants tested within specific genes. The number of dose levels was also not associated with the detection of a statistical finding (*p* = 0.088; Wilcoxon rank sum test). Lastly, between 31 and 50% of studies on major phase I trial endpoints failed to provide any statistical analysis, typically due to low genetic variability or low patient numbers precluding an analysis.

To our knowledge, the present analysis is the first to assemble and analyze several aspects of all published phase I clinical trials including pharmacogenetics in oncology. It is consistent with previous suggestions that pharmacogenomics assessments may need to be delayed for better powered phase II or III clinical trials in most circumstances [3]. Additionally, the endpoints of phase I studies examined in this review are a function of many factors that may reduce the penetrance of each genetic variant, such as age, race, sex, polypharmacy, prior therapy, and other factors [17]. Rarely are these factors included in multivariate analyses along with genotype despite heterogeneous patient cohorts in spite of a high degree of heterogeneity found in phase I trial designs. Most of these studies also focused on genes that were known to affect ADME-A or pharmacodynamics pathways even though tested variants in these genes did not have a high degree of analytic or clinical validity. Of those that did study well-characterized variants, almost none had sufficient coverage of important pharmacogenetic variants that are known to affect drug disposition. Lastly, it is understandable that pharmacogenetics is often a secondary endpoint of phase I studies, leading to insufficient recruitment to conduct a formal statistical analysis. Low genotype representation, however, can be overcome by including estimates of minor allele frequency in study design, recruiting racial populations in which pharmacogenetic variants are commonly inherited, or including genotyping in inclusion criteria.

It is estimated that variation in genes that affect the pharmacokinetics or pharmacodynamics of medications accounts for approximately 20–30% of drug response variability overall [18]. To account for such variation during drug development, future phase I trials with pharmacogenetics endpoints should ensure that they are conducted with sufficient statistical power and a high degree of preclinical or clinical evidence, leveraging current knowledge about gene function prior to embarking on pharmacogenetics testing.

## 5. Genotype-Directed Dosing Studies

Most genotype-directed dosing studies have tested differential dosing of irinotecan or other SN-38-related medications in patients carrying *UGT1A1* variants [19,20,21,22,23]. Differential dosing for SN-38 was recommended in all studies. Other studies determine the capecitabine dose in patients carrying the 3R/3R genotype in *thymidylate synthase* (*TYMS*) were useful for capecitabine dosing [24], the dose of ocaratuzumab in patients carrying FC-gamma receptor IIIa (FCGR3A) variants [25], or whether batracyclin could be administered to those carrying slow acetylator phenotypes in *N-acetyl transferase 2* (*NAT2*) in order to ensure low plasma concentration of a toxic metabolite [26]. In every case, these studies had a wealth of preclinical and/or prior clinical evidence to justify attempts to stratify dosing based on genotype [26,27,28,29].

All genotype-directed Phase I studies in irinotecan only examined UGT1A1*28, a polymorphism in the *UGT1A1* promoter that alters the length of a critical TATA box. Yet, there are four different possibilities of TATA box repeat length that are associated with decreasing levels of *UGT1A1* expression at *UGT1A1 (TA)n* (rs3064744): (TA)5 (UGT1A1*36), (TA)6 (UGT1A1*1), (TA)7 (UGT1A1*28), and (TA)8 (UGT1A1*37) [30,31]. These variants are also detected with a variety of methods in phase I studies, including fragment sizing, pyrosequencing, PAGE gel sizing, or undisclosed methodology. However, we have demonstrated that many of these methods lead to incorrect UGT1A1 genotyping at this locus, calling the results of many of these studies into question. Decreased UGT1A1 function is also associated with UGT1A1*6 and UGT1A1*27 [32,33,34,35,36], which were not tested in these studies.

Study design complications are also apparent in other genotype-directed studies. For example, the study examining *TYMS* genotyping examined the *TYMS* gene enhancer region (*TSER*) 2R/3R (rs45445694) and slow accrual resulted in only 5 patients with *TSER* 2R/2R + 2R/3R genotypes being recruited before this arm of the study was closed. Thus, no dosing guidelines were provided for this group of patients, and only one adverse event was reported [24]. Moreover, this study did not probe a well-characterized cysteine substitution in *TYMS* (rs2853542), nor did it evaluate an insertion/deletion polymorphism in the 3′ UTR (rs16430) that is associated with reduced *TYMS* transcription [28]. Patients who harbored the *TSER* 3R genotype may have then been treated at standard dosing in the presence of other allelic variants that may have influenced pharmacokinetics and toxicity. Thus, even though genotype-directed studies are better powered to answer scientific questions about gene–drug interactions, they too may be confounded by inaccurate and/or incomplete genotyping and limited statistical power.

## 6. Frequently Tested Classes of Anticancer Agents

### 6.1. Topoisomerase Inhibitors

#### 6.1.1. Irinotecan, SN38, and Other Formulations Thereof (PEP02, EZM-2208, NK012)

A total of seventeen phase I studies have been published examining irinotecan pharmacogenetics, although several studies compared multiple endpoints to genotype. Every one of these studies includes UGT1A1*28, although several other *UGT1A1* alleles have been studied: UGT1A1*6, UGT1A1*27, UGT1A1*36, UGT1A1*37, and UGT1A1*60. Eight of these studies did not offer a formal statistical analysis, and eight other studies found no relationship between *UGT1A1* alleles and pharmacokinetics (*n* = 2), toxicity (*n* = 2), response (*n* = 1), disease progression (*n* = 2), or survival (*n* = 1). Two studies found UGT1A1*28 was associated with inter-individual variation in pharmacokinetics [37,38] and two did not [39,40]. Three studies found UGT1A1*6 and/or UGT1A1*28 were associated with toxicity [37,40,41] and two did not [38,42]. No relationship between response or survival and any genotype was determined [42,43]. Others have evaluated variants in *ABCB1*, *CYP3A4*, *CYP3A5*, *UGT1A6*, *UGT1A7*, and *UGT1A9*; however, only one study found UGT1A6 phenotype status was related to toxicity [37]. As stated previously, some Phase I studies have studied differential dosing in patients with different *UGT1A1* allelic variants [19,20,21,22,23]. Eight studies provided no formal statistical analysis for an association between *UGT1A1* genotypes and clinical data derived from phase I studies [19,44,45,46,47,48].

#### 6.1.2. Other Topoisomerase Inhibitors (Anthracyclines, Batracyclin, Amino- and Nitro-Camptothecin Derivatives, TAS-103, Topotecan, TP300)

Despite several studies evaluating pharmacogenetic variants in anthracyclines [49], only two studies have evaluated the influence such variants on the toxicity and response in this class of agents. The first study evaluated amrubicin, finding no evidence that a single variant in *NQO1* (609C > T) influences toxicity or response [50]. No formal statistical analysis was conducted for another study that evaluated SNPs in *ABCB4*, *ABCC1*, *CBRR1*, *CBR3*, *FMAO2*, *HNMT*, *SLC10A2*, *SLC28A3*, and *UGT1A6* in relation to doxorubicin toxicity [51].

One study tested two camptothecin derivatives (9-amino-camptothecin and 9-nitro-camptothecin) in a phase I study that compared variants in efflux transporters in relation to pharmacokinetics and toxicity. This study found that a variant in *ABCG2* (Q141K; rs2231142) was associated with aminocamptothecin dose-normalized AUC but not toxicity [52]. A study of topotecan found no relationship between variants in *CYP3A4*, *CYP3A5*, *UGT1A1*, *ABCG2*, and *ABCB1* and topotecan pharmacokinetics [53]. A study evaluating UGT1A1*28 and TAS-103 pharmacokinetics did not conduct a formal statistical analysis [54]. A genotype-directed dosing study in NAT2 slow acetylators was conducted for batracyclin, a topoisomerase I/II inhibitor. A dose was selected for NAT2 slow acetylators, who are at lower risk of exposure to a toxic batracyclin metabolite [26]. Lastly, one study evaluated several variants in drug metabolizing enzymes and AOX1 in relation to TP300 treatment, but this study offered no formal statistical comparison [55].

## 7. Antimetabolites

### 7.1. Capecitabine and 5-FU

Five studies have evaluated capecitabine toxicity and response, one of which also evaluated genotype-directed dosing. A polymorphism in *CDA* (79A > C) was associated with the development of hematologic toxicity in one study and diarrhea in another [56,57]. These studies also examined variants in *DPYD*, *ENOSF1*, *GSTP1*, *MTHFR*, and *TYMS* with no statistical differences in the development of capecitabine toxicity. Another study tested whether variants in *CDA*, *DPYD*, *GSTP1*, and *TYMS* were associated with capecitabine response in patients with anal cancer, finding no relationship [56]. Two studies evaluated *MTHFR* and *TYMS* variants in patients treated with 5-FU with no formal statistical analysis offered [58,59]. A single genotype-directed study evaluated differential dosing of capecitabine in patients with variants in *TSER* 2R/3R genotypes, as was mentioned previously [24].

### 7.2. Pemetrexed, Ralitrexed, and Pralatrexate

Pemetrexed pharmacogenomics has been frequently studied in the Phase I setting. Three studies evaluated variants in *FPGS*, *GGH*, *GIF*, *MTHFR*, *SLC19A1*, and *TYMS* in relation to pemetrexed toxicity and response, finding no relationships [60,61,62]. Conflicting evidence for a relationship between *MTHFR* 1298A > C (rs1801131) and disease progression or overall survival on pemetrexed in head and neck cancer or various solid tumors has been presented [60,61]. No relationship was found for other variants in *MTHFR* and *TYMS* in these studies.

Ralitrexed and pralatrexate are poorly studied. A single study examined the *MTHFR* 667C > T (rs1801133) in relation to ralitrexed toxicity, finding that this variant was associated with overall toxicity [63]. Another study evaluated this variant, *MTHFR* 1298A > C, and the *TYMS* 2R/3R repeat polymorphism (rs45445694) in relation to pralatrexate toxicity, finding no relationship [64].

### 7.3. Gemcitabine and LY2334737 (Oral Gemcitabine Formulation)

Three studies have focused on gemcitabine therapy in the phase I setting. One evaluated LY2334737 toxicity, finding that SNPs in *CDA* (rs818202) and the *HLA* complex (rs3096691) were associated with the development of hepatotoxicity [65]. The other two studies either did not disclose the specific variants in the genes they tested [66] or did not conduct a formal statistical analysis [67].

### 7.4. Other Antimetabolites (S-1, OSI7904L)

S-1 is an oral fluoropyrimidine that combines tegafur with a DPYD inhibitor, 5-cholor-2,24-dihydroxypyridine, and an orotate phosphoribosyl transferase inhibitor, potassium oxonate [68]. A single study evaluated *CYP2A6* variants in relation to S-1 pharmacokinetics, finding that CYP2A6*4, *7 and *9 were associated with a lower metabolic ratio of S-1 (i.e., the exposure ratio of 5-FU to tegafur) [39].

OSI-7904L is a liposomal formulation of a thymidylate synthase inhibitor that noncompetitively inhibits thymine nucleotide synthesis [69]. Two studies examined the *TYMS* 2R/3R repeat (rs45445694) and/or the 3R G/C (rs45445694) variant and found no association with these variants and OSI-7904L toxicity or response [70,71]. A third study detected the same polymorphisms in addition to *MTHFR* 677C > T (rs1081133) but did not conduct a formal statistical analysis [69].

## 8. Antiangiogenic Therapies

Six studies have evaluated whether pharmacogenomics influences Phase I studies of antiangiogenesis agents. A single study evaluated whether variants in three drug efflux transporters were associated with telatinib pharmacokinetics and whether variants in *FLT4* and *VEGFR2* were associated with the development telatinib toxicity; however, no association was detected [72]. Another study found a variant in *VEGFA* (rs833061) was associated with the development of high-grade neutropenia in those treated with pazopanib [62]. Another study evaluating pazopanib pharmacogenetics found CYP3A4*22 carriers had lower pazopanib clearance, whereas variants in *ABCB1*, and *ABCG2* were not related to pazopanib PK [73]. Progression and overall survival following sorafenib has also been examined in the Phase I setting for those with various solid tumors or pancreatic cancer [74,75]. A variant in *VEGFA* (-899GG) was associated with PFS of sorafenib, and two variants were associated with OS (-1154AA and -7TT), although not consistently between the two studies. Two other studies genotyped a wide variety of SNPs in several genes with a possible relationship to vatalanib or pazopanib pharmacology, but neither study conducted a formal statistical analysis [76,77]. Two studies evaluated bevacizumab response or PFS: The first study found that PFS duration was shorter in those carrying the rs6900017 genotype [78], and the second did not provide a formal statistical analysis of *VEGFA* genotypes versus response in patients treated with both bevacizumab and sorafenib [79].

## 9. Critical Analysis of Phase I Studies Incorporating Frequently Tested Drug Classes vs. Pharmacogenetic Variables

Topoisomerase inhibitors, antimetabolites, and antiangiogenic agents represent 116 of the 206 total comparisons and 49 of 82 studies covered in the present review. Multiple lines of evidence suggest that variants in *UGT1A1* are strong predictors of SN-38 metabolism, pathway variants in folate metabolism (i.e., *TYMS* and *MTHFR*) are commonly associated with antimetabolite therapy efficacy, and pathway variants in angiogenesis affect several VEGFA and VEGFR2 (KDR) inhibitors [27,28,80]. It is not surprising that over half of phase I studies account for variants in these genes. Yet, there is no statistical relationship between the number of studies detecting an association with pharmacogenetic variants in the above studies (22 comparisons detected an association and 57 did not) versus those devoted to testing other gene–drug interactions (18 comparisons detected an association and 39 did not; *p* = 0.70; Fisher’s exact test). Again, phase I studies may not be the best platform to answer scientific questions about the relationship between pharmacogenetic variants and outcomes.

## 10. Conclusions

While many of these phase I trials covered herein were conducted prior to the characterization of the analytical or clinical validation of pharmacogenetic variants, the present review clarifies that even modern phase I studies have design complications that frequently preclude or seriously limit answering scientific questions about inter-individual variability attributed to genetics. The goal of phase I trials is to find a safe dose for phase II studies while simultaneously understanding the pharmacologic and PK properties of agents in humans. While assessment of response is not the goal, many phase I studies try to detect a response signal. Except for studies of molecularly targeted agents, phase I studies in oncology attempt to define the maximum tolerated dose of anticancer agents to maximize the potential for response with acceptable toxicity, resulting in a narrow therapeutic window in which inter-individual variation in toxicity or pharmacokinetics can seriously influence outcomes. Thus, early patient stratification can increase success during early development and is desirable from the standpoints of patient safety, increasing efficacy rates, and mitigating the attrition rate of drug development in oncology.

Phase I trials, however, are not restricted to homogeneous populations with different diseases, prior therapies, comorbidities, and other factors that confound statistical relationships in gene–drug interactions. The majority of phase I studies included herein also included combinations of various medications (48 of 84 studies) that may further confound statistical analysis, and many of them fail to conduct a statistical analysis. Such heterogeneity in small patient populations does not lend itself to hypothesis-free genotyping methods; thus, it is not surprising that Phase I studies most commonly use candidate gene methods. However, coverage of genetic variants is also poor in most of these trials. While small studies often need to avoid multiple comparisons, many of these studies may be confounded by unstudied genetic variation—particularly in genes for which several variants are known to influence gene activity. This detraction of phase I studies is simple to correct by studying activating or deactivating variants to inform gene activity in several genes for which this information is readily available. Multigene technologies, such as Pharmacoscan (formerly the DMET array; Thermo Fisher Scientific), probe multiple variants in well-characterized pharmacogenes and classify these variants into a set of curated phenotypes, but such methods were only used in one study we evaluated [13]. Candidate genes often have poor preclinical or clinical justification for testing in the clinical setting, and candidate gene variants frequently have low analytical/clinical validity in phase I studies. Overall, far fewer than 1% of phase I trials include pharmacogenetics (see methods section). Accounting for these difficulties during study design may make pharmacogenetics testing in phase I studies more routine. Moreover, as the cost for developing oncology agents approximates $2.8 billion United States dollars [85], the expense of early testing of genetic variation is miniscule. Thus, appropriately designed pharmacogenetics testing will likely provide a significant return on significant time and investment required to move oncology agents into humans.

## Figures and Tables

**Table 1:** Important parameters of phase I studies incorporating pharmacogenomics approaches.

Study Endpoints vs. Genotype		*n* =	%
	Toxicity	42	50.0
	Pharmacokinetics	40	47.6
	Response	24	28.6
	Progression-free survival	16	19.0
	Genotype-directed dosing	7	8.3
	Overall survival	5	6.0
	Surrogate marker	5	6.0
	Dose	4	4.8
	Drug interaction	3	3.6
	Radiation	1	1.2
Disease			
	Solid tumors	48	57.1
	Gastrointestinal	7	8.3
	Colorectal	6	7.1
	Breast	3	3.6
	NSCLC	3	3.6
	Pancreatic	3	3.6
	Glioblastoma	2	2.4
	Head and Neck	2	2.4
	Adrenal	1	1.2
	Acute lymophoblastic leukemia	1	1.2
	Acute myelogenous leukemia	1	1.2
	Anal	1	1.2
	Chronic lymphocytic leukemia	1	1.2
	Follicular Lymphoma	1	1.2
	Hepatocellular Carcinoma	1	1.2
	Hematologic	1	1.2
	Neuroblastoma	1	1.2
	Soft Tissue Sarcoma	1	1.2
Number of drugs administered			
	1	36	42.9
	2	29	34.5
	3	19	22.6

**Table 2:** Phase I study design factors categorized by gene-drug pairs and study endpoint.

Gene–Drug Pair		Number of Tested Variants	Number of Patients	Number of Dose Levels	Formal Statistical Comparison?	Association?	Reference
**Studies Including Toxicity (*n* = 115 Gene Comparisons, *n* = 42 Studies)**							
*ABCB1*							
	irinotecan	1	23	2	Y	N	Soepenberg et al. (2005) [1]
	9-aminocamptothecin	3	30	3	Y	N	Zamboni et al. (2006) [2]
	9-nitrocamptothecin	3	30	3	Y	N	Zamboni et al. (2006) [2]
	3-AP	3	19	5	Y	Y	Choi et al. (2010) [3]
	danusertib	3	63	3	Y	N	Steeghs et al. (2011) [4]
	pazopanib	3	16	2	Y	N	Infante et al. (2011) [5]
	lapatinib	3	22	3	Y	N	Deeken et al. (2015) [6]
*ABCB4*							
	doxorubicin	1	20	1	N	N/A	Chugh et al. (2015) [7]
*ABCC1*							
	doxorubicin	1	20	1	N	N/A	Chugh et al. (2015) [7]
*ABCC2*							
	9-aminocamptothecin	1	33	3	Y	N	Zamboni et al. (2006) [2]
	9-nitrocamptothecin	1	33	3	Y	N	Zamboni et al. (2006) [2]
*ABCG2*							
	9-aminocamptothecin	1	28	3	Y	N	Zamboni et al. (2006) [2]
	9-nitrocamptothecin	1	28	3	Y	N	Zamboni et al. (2006) [2]
	pazopanib	1	16	2	Y	N	Infante et al. (2011) [5]
	danusertib	2	63	3	Y	N	Steeghs et al. (2011) [4]
*AURKA*							
	danusertib	2	63	3	Y	N	Steeghs et al. (2011) [4]
*AURKB*							
	danusertib	1	63	3	Y	N	Steeghs et al. (2011) [4]
*CBR1*							
	doxorubicin	1	20	1	N	N/A	Chugh et al. (2015) [7]
*CBR3*							
	doxorubicin	1	20	1	N	N/A	Chugh et al. (2015) [7]
*CDA*							
	capecitabine	1	18	3	Y	Y	Deenen et al. (2013) [8]
	capecitabine	1	34	3	Y	Y	Deenen et al. (2015) [9]
	gemcitabine	1	73	7	Y	Y	Faivre et al. (2015) [10]
*CES2*							
	gemcitabine	1	73	7	Y	N	Faivre et al. (2015) [10]
*Cyclin D1*							
	cetuximab	1	22	3	Y	N	Deeken et al. (2015) [6]
*CYP2C8*							
	pazopanib	2	16	2	Y	N	Infante et al. (2011) [5]
*CYP2C19*							
	tivantinib	2	51	5	Y	Y	Yap et al. (2011) [11]
	tivantinib	2	28	4	N	N/A	Okusaka et al. (2015) [12]
	tivantinib	undisclosed	25	4	N	N/A	Yamamoto et al. (2013) [13]
*CYP3A4*							
	pazopanib	1	16	2	Y	N	Infante et al. (2011) [5]
	irinotecan	3	23	2	Y	N	Soepenberg et al. (2005) [1]
*CYP3A5*							
	vinorelbine	1	24	5	N	N/A	Schott et al. (2006) [14]
	irinotecan	1	23	2	Y	N	Soepenberg et al. (2005) [1]
	17-AAG	1	21	11	N	N/A	Goetz et al. (2005) [15]
	pazopanib	1	16	2	Y	N	Infante et al. (2011) [5]
	lapatinib	3	22	3	Y	N	Deeken et al. (2015) [6]
*DPYD*							
	capeciitabine	2	34	3	Y	N	Deenen et al. (2015) [9]
	capeciitabine	3	18	3	Y	N	Deenen et al. (2013) [8]
*EGF*							
	cetuximab	1	22	3	Y	N	Deeken et al. (2015) [6]
*EGFR*							
	cetuximab	1	22	3	Y	N	Deeken et al. (2015) [6]
*ENOSF1*							
	capeciitabine	1	34	3	Y	Y	Deenen et al. (2015) [9]
*ERBB2*							
	lapatinib	1	22	3	Y	N	Deeken et al. (2015) [6]
*ERCC1*							
	oxaliplatin	1	34	3	Y	Y	Deenen et al. (2015) [9]
	oxaliplatin	undisclosed	16	1	Y	N	Caponigro et al. (2009) [16]
*ERCC2*							
	oxaliplatin	1	34	3	Y	Y	Deenen et al. (2015) [9]
*FcgRIIa*							
	cetuximab	1	22	3	Y	N	Deeken et al. (2015) [6]
*FcgRIIIa*							
	cetuximab	1	22	3	Y	N	Deeken et al. (2015) [6]
	cetuximab	1	23	3	Y	N	McMichael et al. (2019) [17]
*FLT3*							
	danusertib	1	63	3	Y	N	Steeghs et al. (2011) [4]
*FLT4*							
	danusertib	1	63	3	Y	N	Steeghs et al. (2011) [4]
*FMAO2*							
	doxorubicin	1	20	1	N	N/A	Chugh et al. (2015) [7]
*FMO3*							
	danusertib	3	63	3	Y	N	Steeghs et al. (2011) [4]
*FPGS*							
	pemetrexed	1	16	2	Y	N	Infante et al. (2011) [5]
*GGH*							
	pemetrexed	2	16	2	Y	N	Infante et al. (2011) [5]
*GIF*							
	pemetrexed	1	16	2	Y	N	Infante et al. (2011) [5]
*GSTP1*							
	capeciitabine	1	18	3	Y	N	Deenen et al. (2013) [8]
	oxaliplatin	1	34	3	Y	Y	Deenen et al. (2015) [9]
*GSTT1*							
	oxaliplatin	1	34	3	Y	N	Deenen et al. (2015) [9]
*HLA*							
	gemcitabine	1	73	13	Y	N	Faivre et al. (2015) [10]
*HNMT*							
	doxorubicin	1	20	1	N	N/A	Chugh et al. (2015) [7]
*MTHFR*							
	ralitrexed	1	33	9	Y	Y	Stevenson et al. (2001) [18]
	5-FU	1	24	5	N	N/A	Veronese et al. (2004) [19]
	capeciitabine	1	34	3	Y	N	Deenen et al. (2015) [9]
	pemetrexed	2	16	2	Y	N	Infante et al. (2011) [5]
	pralatrexate	2	27	5	Y	N	Grem et al. (2015) [20]
	pemetrexed	3	32	3	Y	N	Argiris et al. (2011) [21]
*NQO1*							
	17-AAG	1	21	11	N	N/A	Goetz et al. (2005) [15]
	amrubicin	1	36	4	Y	N	Jalal et al. (2017) [22]
*SLC10A2*							
	doxorubicin	1	20	1	N	N/A	Chugh et al. (2015) [7]
*SLC19A1*							
	pemetrexed	1	16	2	Y	N	Infante et al. (2011) [5]
*SLC28A1*							
	gemcitabine	1	73	7	Y	N	Faivre et al. (2015) [10]
*SLC28A3*							
	doxorubicin	1	20	1	N	N/A	Chugh et al. (2015) [7]
*RET*							
	danusertib	2	63	3	Y	N	Steeghs et al. (2011) [4]
*TYMS*							
	OSI-7904L	1	31	8	Y	N	Beutel et al. (2005) [23]
	capeciitabine	1	34	3	Y	Y	Deenen et al. (2015) [9]
	pralatrexate	1	27	5	Y	N	Grem et al. (2015) [20]
	Capeciitabine *	1	23	4	Y	N	Soo et al. (2016) [24]
	OSI-7904L	2	15	3	Y	N	Clamp et al. (2008) [25]
	pemetrexed	2	32	3	Y	N	Argiris et al. (2011) [21]
	capeciitabine	2	18	3	Y	N	Deenen et al. (2013) [8]
	pemetrexed	2	16	2	Y	N	Infante et al. (2011) [5]
*UGT1A1*							
	flavopiridol	1	49	9	Y	N	Zhai et al. (2003) [26]
	irinotecan	1	23	2	Y	N	Soepenberg et al. (2005) [1]
	irinotecan	1	28	3	Y	N	Font et al. (2008) [27]
	irinotecan	1	45	1	Y	N	Denlinger et al. (2009) [28]
	3-AP	1	19	5	N	N/A	Choi et al. (2010) [3]
	nilotinib	1	111	9	Y	Y	Singer et al. (2007) [29]
	pazopanib	1	16	2	Y	N	Infante et al. (2011) [5]
	gemcitabine	1	73	13	Y	N	Faivre et al. (2015) [10]
	alisertib	1	22	1	Y	N/A	DuBois et al. (2016) [30]
	irinotecan	1	22	1	Y	N/A	DuBois et al. (2016) [30]
	SN-38 *	1	39	7	N	N/A	Burris et al. (2016) [31]
	irinotecan	1	31	2	Y	Y	Federico et al. (2020) [32]
	irinotecan *	1	50	3	N	N/A	Joshi et al. (2020) [33]
	irinotecan *	2	27	4,2	Y	Y	Hazama et al. (2010) [34]
	irinotecan	2	37	3	Y	Y	Yamamoto et al. (2009) [35]
	irinotecan	2	11	3	N	N/A	Chang et al. (2015) [36]
	irinotecan	2	16	4	N	N/A	Chiang et al. (2016) [37]
	irinotecan	2	35	2	Y	N	Ishiguro et al. (2017) [38]
	irinotecan	2	35	2	N	N/A	Yoshino et al. (2017) [39]
	SN-38	3	39	6	N	N/A	Kurzrock et al. (2012) [40]
	irinotecan	3	10	2	N	N/A	Doi et al. (2015) [41]
	belinostat	3	25	4	Y	Y	Goey et al. (2016) [42]
	bortezomib	undisclosed	16	N/A	Y	N	Caponigro et al. (2009) [16]
*UGT1A6*							
	doxorubicin	1	20	1	N	N/A	Chugh et al. (2015) [7]
	irinotecan	3	45	1	Y	Y	Denlinger et al. (2009) [28]
*UGT1A7*							
	irinotecan	4	45	1	Y	N	Denlinger et al. (2009) [28]
*UGT1A9*							
	irinotecan	1	45	1	Y	N	Denlinger et al. (2009) [28]
*VEGFA*							
	pazopanib	2	16	2	Y	Y	Infante et al. (2011) [5]
	teletanib	3	33	7	Y	N	Steeghs et al. (2011) [43]
*VEGFR2*							
	pazopanib	2	16	2	Y	N	Infante et al. (2011) [5]
	danusertib	5	63	3	Y	N	Steeghs et al. (2011) [4]
*XPD*							
	oxaliplatin	1	15	3	Y	N	Clamp et al. (2008) [25]
	cisplatin	2	28	3	Y	N	Font et al. (2008) [27]
*XRCC1*							
	oxaliplatin	undisclosed	16	1	Y	N	Caponigro et al. (2009) [16]
*XRCC3*							
	cisplatin	2	28	3	Y	N	Font et al. (2008) [27]
**Studies Including Response or Progression-Free Survival (*n* = 76 Gene Comparisons, *n* = 32 Studies)**							
*ABCB1*							
	lapatinib	3	22	3	Y	N	Deeken et al. (2015) [6]
	paclitaxel	3	27	3	Y	N	Chiorean et al. (2020) [44]
*APRIL*							
	atacicept	3	19	6	Y	Y	Kofler et al. (2012) [45]
*BCMA*							
	atacicept	2	19	6	Y	N	Kofler et al. (2012) [45]
*Cyclin D1*							
	cetuximab	1	22	3	Y	N	Deeken et al. (2015) [6]
*CDA*							
	capecitabine	1	34	3	Y	N	Deenen et al. (2015) [9]
	gemcitabine	undisclosed	89	1	Y	N	Philip et al. (2014) [46]
*CYP2C8*							
	paclitaxel	1	27	3	Y	N	Chiorean et al. (2020) [44]
*CYP24A1*							
	calcitriol	28	20	4	Y	Y	Ramnath et al. (2013) [47]
*CYP3A4*							
	paclitaxel	1	27	3	Y	N	Chiorean et al. (2020) [44]
*CYP3A5*							
	lapatinib	3	22	3	Y	N	Deeken et al. (2015) [6]
	paclitaxel	3	27	3	Y	N	Chiorean et al. (2020) [44]
*DPYD*							
	capeciitabine	2	34	3	Y	N	Deenen et al. (2015) [9]
*EGF*							
	cetuximab	1	22	3	Y	N	Deeken et al. (2015) [6]
	erlotinib	undisclosed	89	1	Y	N	Philip et al. (2014) [46]
*EGFR*							
	cetuximab	1	22	3	Y	N	Deeken et al. (2015) [6]
	erlotinib	undisclosed	89	1	Y	N	Philip et al. (2014) [46]
*ENOSF1*							
	capeciitabine	1	34	3	Y	N	Deenen et al. (2015) [9]
*ERBB2*							
	lapatinib	1	22	3	Y	N	Deeken et al. (2015) [6]
*ERCC1*							
	oxaliplatin	undisclosed	16	1	Y	N	Caponigro et al. (2009) [16]
	oxaliplatin	1	34	3	Y	N	Deenen et al. (2015) [9]
*ERCC2*							
	oxaliplatin	1	34	3	Y	N	Deenen et al. (2015) [9]
*FcgRIIa*							
	cetuximab	1	22	3	Y	Y	Deeken et al. (2015) [6]
	erlotinib	undisclosed	89	1	Y	N	Philip et al. (2014) [46]
*FcgRIIIa*							
	cetuximab	1	22	3	Y	N	Deeken et al. (2015) [6]
	cetuximab	1	23	3	Y	N	McMichael et al. (2019) [17]
	octratuzumab *	1	50	5	Y	Y	Ganjoo et al. (2015) [48]
	erlotinib	undisclosed	89	1	Y	N	Philip et al. (2014) [46]
*FLT1*							
	sorafenib	1	27	3	Y	N	Chiorean et al. (2020) [44]
*GSTP1*							
	oxaliplatin	1	34	3	Y	N	Deenen et al. (2015) [9]
*GSTT1*							
	oxaliplatin	1	34	3	Y	N	Deenen et al. (2015) [9]
*HER2*							
	trastuzumab	5	56	12	N	N/A	Falchook et al. (2015) [49]
*IFNgamma*							
	trastuzumab, IL12	1	15	5	N	N/A	Parihar et al. (2004) [50]
*IGF1*							
	erlotinib	undisclosed	89	1	Y	Y	Philip et al. (2014) [46]
*IL6*							
	trastuzumab, IL12	2	15	5	N	N/A	Parihar et al. (2004) [50]
*IL8*							
	erlotinib	undisclosed	89	1	Y	N	Philip et al. (2014) [46]
*IL10*							
	trastuzumab, IL12	3	15	5	N	N/A	Parihar et al. (2004) [50]
*MTHFR*							
	capeciitabine	1	34	3	Y	N	Deenen et al. (2015) [9]
	OSI-7904L	1	30	4	N	N/A	Ricart et al. (2008) [51]
	pemetrexed	2	89	3	Y	N	Chen et al. (2010) [52]
	pemetrexed	3	32	3	Y	N	Argiris et al. (2011) [21]
*NAT2*							
	JPH203	10	17	5	N	N/A	Okano et al. (2020) [53]
*NQO1*							
	amrubicin	1	36	4	Y	N	Jalal et al. (2017) [22]
*ODC*							
	DFMO	2	21	4	Y	N	Saulnier Sholler et al. (2015) [54]
*PARP1*							
	olaparib	1	45	6	N	N/A	Lee et al. (2014) [55]
*RRM1*							
	gemcitabine	undisclosed	89	1	Y	N	Philip et al. (2014) [46]
*TACI*							
	atacicept	5	19	6	Y	Y	Kofler et al. (2012) [45]
*TGFB*							
	trastuzumab, IL12	2	15	5	N	N/A	Parihar et al. (2004) [50]
*TNFalpha*							
	trastuzumab, IL12	1	15	5	N	N/A	Parihar et al. (2004) [50]
*TUBB*							
	ABT-571	8	32	6	N	N/A	Yee et al. (2005) [56]
*TYMS*							
	5-FU	1	28	4	N	N/A	Wright et al. (2005) [57]
	OSI-7904L	1	31	8	Y	N	Beutel et al. (2005) [23]
	capeciitabine *	1	23	4	Y	N	Soo et al. (2016) [24]
	capeciitabine	1	34	3	Y	N	Deenen et al. (2015) [9]
	OSI-7904L	2	15	3	Y	N	Clamp et al. (2008) [25]
	OSI-7904L	2	30	4	N	N/A	Ricart et al. (2008) [51]
	pemetrexed	2	32	3	Y	N	Argiris et al. (2011) [21]
*UGT1A1*							
	irinotecan	1	30	4	Y	N	Wright et al. (2005) [57]
	irinotecan	1	28	3	Y	N	Font et al. (2008) [27]
	irinotecan *	1	44	5,4	Y	Y	Toffoli et al. (2010) [58]
	SN-38 *	1	39	7	N	N/A	Burris et al. (2016) [31]
	irinotecan *	1	50	3	N	N/A	Joshi et al. (2020) [33]
	bortezomib	undisclosed	16	N/A	Y	N	Caponigro et al. (2009) [16]
	irinotecan	2	35	2	Y	N	Ishiguro et al. (2017) [38]
*VEGFA*							
	sorafenib, bevacizumab	4	115	4	N	N/A	Falchook et al. (2015) [59]
	sorafenib	4	27	3	Y	N	Chiorean et al. (2014) [60]
	sorafenib	7	27	3	Y	N	Chiorean et al. (2020) [44]
	bevacizumab	9	110	3	Y	Y	Sen et al. (2014) [61]
*VEGFR2*							
	sorafenib	3	27	3	Y	Y	Chiorean et al. (2014) [60]
	vatalanib	30	10	4	N	N/A	Gerstner et al. (2011) [62]
*XPD*							
	oxaliplatin	1	15	3	Y	N	Clamp et al. (2008) [25]
	oxaliplatin	1	30	2	N	N/A	Ricart et al. (2008) [51]
	cisplatin	2	28	3	Y	N	Font et al. (2008) [27]
*XRCC1*							
	carboplatin	2	45	6	N	N/A	Lee et al. (2014) [55]
	oxaliplatin	undisclosed	16	1	Y	N	Caponigro et al. (2009) [16]
*XRCC3*							
	cisplatin	2	28	3	Y	Y	Font et al. (2008) [27]
**Studies Including Pharmacokinetics (*n* = 90 Gene Comparisons, *n* = 40 Studies)**							
*ABCB1*							
	irinotecan	1	23	2	Y	N	Soepenberg et al. (2005) [1]
	pazopanib	1	94	5	Y	N	Bins et al. (2019) [63]
	lapatinib	2	24	3	Y	N	Thiessen et al. (2010) [64]
	erlotinb	2	88	2	Y	Y	White-Koning et al. (2011) [65]
	9-aminocamptothecin	3	30	3	Y	N	Zamboni et al. (2006) [2]
	9-nitrocamptothecin	3	30	3	Y	N	Zamboni et al. (2006) [2]
	paclitaxel	3	10	3	N	N/A	Veltkamp et al. (2007) [66]
	danusertib	3	63	3	Y	N	Steeghs et al. (2011) [4]
	paclitaxel	3	27	3	N	N/A	Chiorean et al. (2020) [44]
	teletanib	4	33	7	Y	N	Steeghs et al. (2011) [43]
*ABCC1*							
	teletanib	4	33	7	Y	N	Steeghs et al. (2011) [43]
*ABCC2*							
	9-aminocamptothecin	1	33	3	Y	N	Zamboni et al. (2006) [2]
	9-nitrocamptothecin	1	33	3	Y	N	Zamboni et al. (2006) [2]
*ABCG2*							
	9-aminocamptothecin	1	28	3	Y	Y	Zamboni et al. (2006) [2]
	9-nitrocamptothecin	1	28	3	Y	N	Zamboni et al. (2006) [2]
	erlotinib	1	88	2	Y	Y	White-Koning et al. (2011) [65]
	salazosulfapyridine	1	15	3	N	N/A	Otsubo et al. (2017) [67]
	danusertib	2	63	3	Y	N	Steeghs et al. (2011) [4]
	teletanib	2	33	7	Y	N	Steeghs et al. (2011) [43]
	pazopanib	2	94	5	Y	N	Bins et al. (2019) [63]
	lapatinib	undisclosed	24	3	Y	N	Thiessen et al. (2010) [64]
*AOX1*							
	TP300	1	32	7	N	N/A	Anthoney et al. (2012) [68]
*AURKA*							
	danusertib	2	63	3	Y	N	Steeghs et al. (2011) [4]
*AURKB*							
	danusertib	1	63	3	Y	N	Steeghs et al. (2011) [4]
*CDA*							
	oral gemcitabine (LY2334737)	1	13	3	N	N/A	Yamamoto et al. (2013) [69]
*CES2*							
	oral gemcitabine (LY2334737)	1	13	3	N	N/A	Yamamoto et al. (2013) [69]
*CYP24A1*							
	calcitriol	28	20	4	Y	N	Ramnath et al. (2013) [47]
*CYP2A6*							
	S-1	4	23	3	Y	Y	Park et al. (2013) [70]
	letrozole	8	22	2	Y	Y	Tanii et al. (2011) [71]
*CYP2C19*							
	E7070	2	21	5	N	N/A	Yamada et al. (2005) [72]
	tivantinib	2	51	5	Y	N	Yap et al. (2011) [11]
	nelfenavir	2	39	2	Y	Y	Kattel et al. (2015) [73]
	tivantinib	2	28	4	N	N/A	Okusaka et al. (2015) [12]
	ibrutinib, voriconazole	61	26	3	N	N/A	de Jong et al. (2018) [74]
	tivantinib	undisclosed	47	8	N	N/A	Yamamoto et al. (2013) [75]
	tivantinib	undisclosed	25	4	N	N/A	Yamamoto et al. (2013) [13]
*CYP2C8*							
	paclitaxel	1	27	3	Y	N	Chiorean et al. (2020) [44]
*CYP2C9*							
	E7070	2	21	5	N	N/A	Yamada et al. (2005) [72]
	abemaciclib	2	44	1	N	N/A	Turner et al. (2020) [76]
*CYP2D6*							
	TP300	2	32	7	N	N/A	Anthoney et al. (2012) [68]
	abemaciclib	12	44	1	N	N/A	Turner et al. (2020) [76]
*CYP3A4*							
	panobinostat	1	14	2	N	N/A	Hamberg et al. (2011) [77]
	pazopanib	1	94	5	Y	Y	Bins et al. (2019) [63]
	paclitaxel	1	27	3	Y	N	Chiorean et al. (2020) [44]
	irinotecan	3	23	2	Y	N	Soepenberg et al. (2005) [1]
	abemaciclib	4	44	1	N	N/A	Turner et al. (2020) [76]
	ibrutinib, erythromycin	51	26	3	N	N/A	de Jong et al. (2018) [74]
	lapatinib	undisclosed	24	3	Y	N	Thiessen et al. (2010) [64]
*CYP3A5*							
	irinotecan	1	23	2	Y	N	Soepenberg et al. (2005) [1]
	17-AAG	1	21	11	N	N/A	Goetz et al. (2005) [15]
	lapatinib	1	24	3	Y	N	Thiessen et al. (2010) [64]
	erlotinib	1	88	2	Y	Y	White-Koning et al. (2011) [65]
	paclitaxel	3	27	3	Y	N	Chiorean et al. (2020) [44]
	panobinostat	4	14	2	N	N/A	Hamberg et al. (2011) [77]
	abemaciclib	5	44	1	N	N/A	Turner et al. (2020) [76]
	ibrutinib, erythromycin	22	26	3	N	N/A	de Jong et al. (2018) [74]
*FLT1*							
	sorafenib	1	27	3	Y	N	Chiorean et al. (2020) [44]
*FLT3*							
	danusertib	1	63	3	Y	N	Steeghs et al. (2011) [4]
*FLT4*							
	danusertib	1	63	3	Y	N	Steeghs et al. (2011) [4]
*FMO3*							
	danusertib	3	63	3	Y	Y	Steeghs et al. (2011) [4]
*NAT2*							
	salazosulfapyridine	4	15	3	N	N/A	Otsubo et al. (2017) [67]
	JPH203	10	17	5	N	N/A	Okano et al. (2020) [53]
*NQO1*							
	17-AAG	1	21	11	N	N/A	Goetz et al. (2005) [15]
	Rh1	1	14	12	N	N/A	Danson et al. (2011) [78]
*RET*							
	danusertib	2	63	3	Y	N	Steeghs et al. (2011) [4]
*TYMS*							
	5-FU	1	28	4	N	N/A	Wright et al. (2005) [57]
*UGT1A1*							
	TAS-103	1	12	1	N	N/A	Ewesuedo et al. (2001) [79]
	flavopiridol	1	49	9	Y	N	Zhai et al. (2003) [26]
	irinotecan	1	23	2	Y	Y	Soepenberg et al. (2005) [1]
	irinotecan	1	30	4	Y	Y	Wright et al. (2005) [57]
	irinotecan	1	45	1	Y	Y	Denlinger et al. (2009) [28]
	irinotecan *	1	44	5,4	Y	Y	Toffoli et al. (2010) [58]
	TP300	1	32	7	N	N/A	Anthoney et al. (2012) [68]
	topotecan	1	29	3	Y	N	Stewart et al. (2014) [80]
	alisertib	1	22	1	Y	N/A	DuBois et al. (2016) [30]
	irinotecan	1	22	1	Y	N/A	DuBois et al. (2016) [30]
	irinotecan	2	37	3	Y	N	Yamamoto et al. (2009) [35]
	irinotecan *	2	27	4,2	Y	Y	Hazama et al. (2010) [34]
	irinotecan	2	11	3	N	N/A	Chang et al. (2015) [36]
	irinotecan	2	16	4	N	N/A	Chiang et al. (2016) [37]
	irinotecan	3	23	4	Y	N	Park et al. (2013) [70]
	irinotecan *	3	18	unknown	unknown	unknown	Takano et al. (2013) [81]
	belinostat	3	25	4	Y	Y	Goey et al. (2016) [42]
*UGT1A6*							
	irinotecan	3	45	1	Y	N	Denlinger et al. (2009) [28]
	irinotecan	4	23	4	Y	N	Park et al. (2013) [70]
*UGT1A7*							
	irinotecan	4	45	1	Y	N	Denlinger et al. (2009) [28]
	irinotecan	4	23	4	Y	N	Park et al. (2013) [70]
*UGT1A9*							
	irinotecan	1	45	1	Y	N	Denlinger et al. (2009) [28]
*VEGFA*							
	sorafenib	7	27	3	Y	N	Chiorean et al. (2020) [44]
*VEGFR2*							
	danusertib	5	63	3	Y	N	Steeghs et al. (2011) [4]
**Other Studies (*n* = 3 Studies)**							
*MTD and toxicity in NAT2 slow acetylators*							
*NAT2*							
	batracyclin	11	31	4	N/A	N/A	Kummar et al. (2013) [82]
*Dose escalation only evaluating genotypes in discontinued patients*							
	pazopanib/paclitaxel	3	28	undisclosed	N/A	N/A	Kendra et al. (2013) [83]
*FcgRIIIa (no variants identified)*							
	cetuximab	3	22	1	N/A	N/A	Bertino et al. (2016) [84]

* Genotype-directed study.
